# The Catalytic Subunit of the System L1 Amino Acid Transporter (*Slc7a5*) Facilitates Nutrient Signalling in Mouse Skeletal Muscle

**DOI:** 10.1371/journal.pone.0089547

**Published:** 2014-02-26

**Authors:** Nadège Poncet, Fiona E. Mitchell, Adel F. M. Ibrahim, Victoria A. McGuire, Grant English, J. Simon C Arthur, Yun-Bo Shi, Peter M. Taylor

**Affiliations:** 1 Division of Cell Signalling and Immunology, College of Life Sciences, University of Dundee, Dundee, United Kingdom; 2 Medical Research Council Protein Phosphorylation and Ubiquitylation Unit, College of Life Sciences, University of Dundee, Dundee, United Kingdom; 3 Division of Molecular Microbiology, College of Life Sciences, University of Dundee, Dundee, United Kingdom; 4 Section on Molecular Morphogenesis, Program in Cellular Regulation and Metabolism (PCRM), NICHD, NIH, Bethesda, Maryland, United States of America; Tohoku University, Japan

## Abstract

The System L1-type amino acid transporter mediates transport of large neutral amino acids (LNAA) in many mammalian cell-types. LNAA such as leucine are required for full activation of the mTOR-S6K signalling pathway promoting protein synthesis and cell growth. The SLC7A5 (LAT1) catalytic subunit of high-affinity System L1 functions as a glycoprotein-associated heterodimer with the multifunctional protein SLC3A2 (CD98). We generated a floxed *Slc7a5* mouse strain which, when crossed with mice expressing Cre driven by a global promoter, produced *Slc7a5* heterozygous knockout (*Slc7a5*+/−) animals with no overt phenotype, although homozygous global knockout of *Slc7a5* was embryonically lethal. Muscle-specific (MCK Cre-mediated) *Slc7a5* knockout (MS-*Slc7a5*-KO) mice were used to study the role of intracellular LNAA delivery by the SLC7A5 transporter for mTOR-S6K pathway activation in skeletal muscle. Activation of muscle mTOR-S6K (Thr389 phosphorylation) *in vivo* by intraperitoneal leucine injection was blunted in homozygous MS-*Slc7a5*-KO mice relative to wild-type animals. Dietary intake and growth rate were similar for MS-*Slc7a5*-KO mice and wild-type littermates fed for 10 weeks (to age 120 days) with diets containing 10%, 20% or 30% of protein. In MS-*Slc7a5*-KO mice, Leu and Ile concentrations in gastrocnemius muscle were reduced by ∼40% as dietary protein content was reduced from 30 to 10%. These changes were associated with >50% decrease in S6K Thr389 phosphorylation in muscles from MS-*Slc7a5*-KO mice, indicating reduced mTOR-S6K pathway activation, despite no significant differences in lean tissue mass between groups on the same diet. MS-*Slc7a5*-KO mice on 30% protein diet exhibited mild insulin resistance (*e.g*. reduced glucose clearance, larger gonadal adipose depots) relative to control animals. Thus, SLC7A5 modulates LNAA-dependent muscle mTOR-S6K signalling in mice, although it appears non-essential (or is sufficiently compensated by *e.g*. SLC7A8 (LAT2)) for maintenance of normal muscle mass.

## Introduction

Amino acids (AAs) are both raw materials and fuel for protein synthesis and hence for growth and development of the human body. Large neutral amino acids (LNAA), especially leucine, also exert a permissive effect on the intracellular mTOR-S6K (mTORC1) cell-signalling pathway which promotes net protein synthesis and cell growth (*e.g.* [1], [2] for review). It is clear from previous findings [3], [4], [5], [6] that the System L1 (leucine-preferring) AA transporter is a key early player in transduction of an extracellular LNAA stimulus to signalling pathways such as mTORC1 and may therefore have functional importance for control of cell and body growth, as well as in the coupling of growth and survival signals (*e.g.* from growth factors). The System L1 transporter is an Na^+^-independent obligatory exchanger of LNAA (substrates include aromatic and branched-chain amino acids and iodothyronines such as T_3_) which is composed of two protein subunits: a catalytic LNAA permease (either SLC7A5/LAT1 or SLC7A8/LAT2 aka CD98lc) and a regulatory glycoprotein (SLC3A2; also known as 4F2hc or CD98hc). SLC7A5 transport characteristics conform to that of System L1 transport, the SLC7A5 System L1 transporter isoform is expressed in many tissues including skeletal muscle, adipose, placenta and brain. It is therefore likely to be a major contributor to cellular and whole-body fluxes of LNAA, especially given that SLC7A5 substrates include 6 of 8 dietary-essential AAs. The expression of *Slc7a5* more closely and selectively correlates with System L1 transport function than expression of *Slc3a2*; indeed the latter associates with several different SLC7 permeases and other cell surface proteins [7].

SLC7A5 is able to modulate intracellular LNAA concentrations (and consequently signalling downstream of intracellular AA sensors) through a process of coupled AA transport, for example with the Na^+^-coupled SLC38A2 (SNAT2) or SLC1A5 (ASCT2) transporters which pump AA such as glutamine into cells as an exchange substrate for essential LNAA entry through SLC7A5 [5], [8]. Expression of *Slc7a5*, *Slc3a*2 and *Slc38a2* in skeletal muscle is rapidly (though transiently) upregulated following essential AA ingestion in humans [9] and is associated with the muscle protein anabolic response. Such observations highlight an increasing recognition that these AA transporters may be limiting components for generation of an anabolic response to dietary protein, in terms of both substrate supply and an activating signal for mRNA translation [9], [10]. The HIF2α pathway increases mTORC1 activity by directly upregulating the expression of *Slc7a5* [6] and a variety of other signalling inputs influence the effectiveness of LNAA as mTORC1 activators (eg. [11], [12], [13]). The realization that essential AA (EAA) such as leucine are required for full activation of mTORC1 signalling downstream of insulin and other growth factors has prompted numerous recent studies on the possible use of dietary leucine as an adjunct treatment for insulin resistance related to obesity (e.g. [14], [15], [16]).

In this context, the relationship between the SLC7A5 LNAA transporter function and anabolic signalling *in vivo* requires closer scrutiny. To this end, we have generated a transgenic mouse line in which the gene encoding *Slc7a5* includes LoxP sites, flanking a 1855bp region of the *Slc7a5* gene. The flanked region includes the transcription start site and 1^st^ exon (*Slc7a5*-Flox). These LoxP sites are targets of recombinase which excises this part of the gene to knockout *Slc7a5* expression in mice containing both *Slc7a5*-Flox and Cre transgenes. Here, we investigate the functions of SLC7A5 in mouse physiology by studying mouse strains harbouring a Cre-LoxP conditional knockout in skeletal muscle, in order to establish the importance of LNAA delivery by SLC7A5 for activation of the mTOR-S6K signalling pathway and its relationship to control of muscle mass *in vivo*.

## Materials and Methods

### Ethics Statement

Mice were housed in animal facilities at the University of Dundee (UK) and in the National Institutes of Health (USA). All animal breeding and experimental procedures received ethics committee approval and were performed under authority of either PPL 60/3455 and 60/4118 (UK) or ASP 07/019 and 10/019 (USA). All procedures necessary for generation of the *Slc7a5*-Flox mouse line were undertaken in the transgenic animal facilities of the University of Dundee, under Home Office PPL 60/2365.

### Generation of Targeting Vector Transgene and Chimeric Mouse

**Figure 1:**
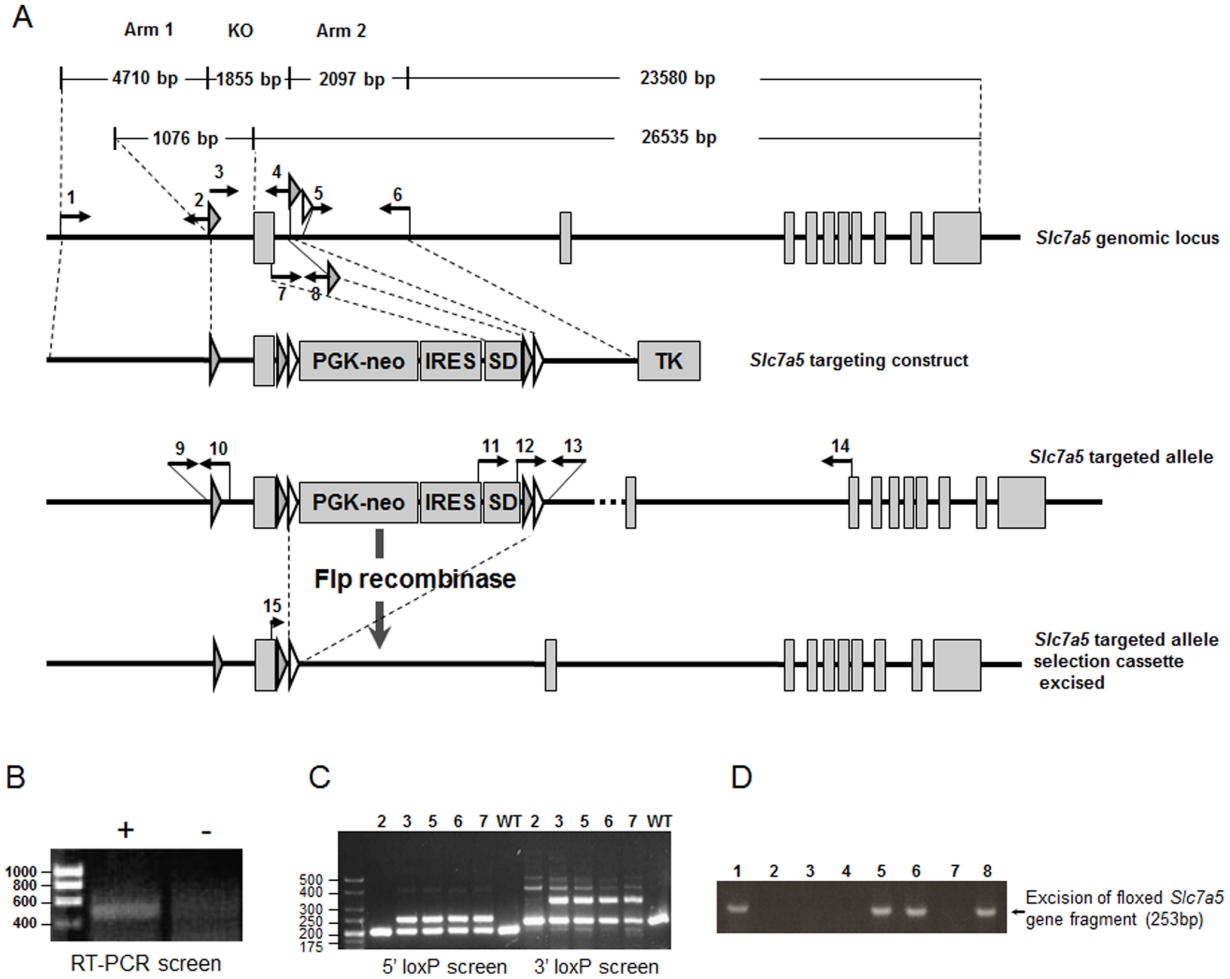
*Slc7a5-*Flox gene construct and mouse genotyping scheme. (A) Graphical representation of the *Slc7a5*-Flox construct used to generate the chimera. Primer locations used for vector assembly and genotyping are indicated and primer sequences shown in Table 1. LoxP sites (grey-filled triangles), FRT sites (white-filled triangles), phosphoglucokinase (PGK) promoter-driven neomycin-resistance gene (PGK-neo) and thymidine kinase gene (TK) are shown. The splice donor (SD) was incorporated into the targeting construct to allow correct splicing of the transcript generated from the PGK promoter only if the targeting construct is inserted within an intron (see B). (B) to (D) Representative PCR genotyping results. (B) RT-PCR screen of RNA from mouse embryonic stem cells using 11–14 primer pair, a 500 bp product would indicate correct joining of the exonic sequence from the splice donor with exon3 of *Slc7a5*. Gel shows RT-PCR for a positive ES cell population (+) or control cells (–). (C) Genotyping PCR for the 5′ loxP site using DNA from C57Bl/6 (WT) or a mixed litter of wild-type (2) and heterozygous Slc7a5 Flox^Neo^ (+/−) (3, 5, 6, 7) mice as a template resulted in either a product of 202 bp or products of 202 and 250 bp, respectively using the 9–10 primer pair. For 3′ loxP site genotyping, PCR using primer pair 12–13 (note primer 12 is duplicated in SD) generated a 230 bp product from wild-type samples or products of 230 and 318 bp from heterozygotes. (D) PCR analysis of genomic DNA from a single mixed litter of heterozygous *Slc7a5*-Flox (2,3,4,7) and *Slc7a5+/−* (1,5,6,8) mice using the 9–13 primer pair which generates a product of 235 bp only with the recombined *Slc7a5* gene lacking the 1855 bp floxed region (which includes exon1).

The *Slc7a5* gene was amplified by PCR in segments from BAC clone RP23-428C21. Restriction sites were incorporated into the primer sets (see Table 1), directing insertion of restriction sites at the end of amplicons. A transgenic targeting construct (Figure 1A) was assembled in sections that included a neomycin-resistance cassette and a herpes simplex thymidine kinase gene. To increase targeting efficiency, a polyA trap neomycin cassette was used in which the neo open reading frame was followed by an IRES sequence and the splice donor sequence from exon1 of the *Slc7a5* gene. The construct was electroporated into E14 mouse embryonic stem (ES) cells and those with homologous recombination of the *Slc7a5* transgene were identified through negative and positive selection using neomycin and glanciclovir, respectively [17]. ES cells were then screened by RT-PCR using primers with binding sites located in the IRES element in the SLC7A5 targeting construct and in exon3 of the *Slc7a5* gene, which is located downstream of the vector insertion site (Figure 1A and 1B). ES cells that expressed the product of the IRES (and therefore harboured the transgene) were selected for implantation into mouse embryos. Chimeric mice which gave germline transmission were crossed with Flpe transgenic mice to remove the neomycin selection cassette.

**Table 1:** Sequences of DNA primers for transgenic construct and for genotyping SLC7A5 transgenic mice.

Oligo	Use	Sequence
1	Homology arm 1, sense, NotI	CTGCGGCCGCGATCATCTTGCCTGTGGTTGGAACTCAAAGTTC
2	Homology arm 1, antisense, BamHI, loxP, SbfI	CTGGATCCATAACTTCGTATAGCATACATTATACGAAGTTATCCTGCAGGGAAAATTTCATTGGTCAAGATAAGTCCAGGAGC
3	KO sense, BamHI	CTGGATCCCAAGCGTACCATCAGCTCAAATTACACAG
4	KO antisense, FseI, NsiI, loxP	CTGGCCGGCCATGCATATAACTTCGTATAGCATACATTATACGAAGTTATCCCACTGAGGTCTCGCGAGGGCTG
5	Homology Arm 2, sense, SpeI, frt	GCACTAGTGAAGTTCCTATTCCGAAGTTCCTATTCTCTAGAAAGTATAGGAACTTCTCGCGGCTCTGACCCCGCGG
6	Homology Arm 2 antisense, AscI	CTGGCGCGCCCCAGGTCAAGTCGACAGCACCC
7	Splice donor, sense, PacI, Kozak	GCTTAATTAAGCCACCATGGCGGAGGTCTACGGCTCGTTGCC
8	Splice donor 3′ SpeI, loxP	GCACTAGTATAACTTCGTATAGCATACATTATACGAAGTTATCCCACTGAGGTCTCGCGAGGGC
9	5′ loxP site screening, sense,	GGCTCCTGGACTTATCTTGACCAATG
10	5′ loxP site screening antisense	AGATAATGTGGTCACACATCTGGAAGGTTC
11	RT-PCR primer, sense	TGCACATGCTTTACATGTGTTTAGTCGAGG
12	3′ loxp screening, sense,	TGAACCATCTCGGCAGTTCCAGGC
13	3′ loxp screening/Neo cassette screening antisense	GTGGTGCTTTGCTGAAGGCAGGG
14	RT-PCR primer, antisense	CAGATTGGTGCCTTCAAAGGACAACTTC
15	Neomycin cassette screening sense	AGCTGGGCACCACCATCTCCAAG
16	Forward primer to amplify FLPe recombinase	CACCTAAGGTCCTGGTTCGTC
17	Reverse primer to amplify FLPe recombinase	CCCAGATGCTTTCACCCTCAC
18	Forward primer to amplify Cre recombinase	AAATGGTTTCCCGCAGAACC
19	Reverse primer to amplify Cre recombinase	TAGCTGGCTGGTGGCAGATG

### Animals

All animals were maintained on a 12/12 h light/dark cycle and had access to food and water *ad libitium*. The standard laboratory diet contained 14–20% (w/w) dietary protein, and the high (30%) medium (20%) and low (10%) w/w crude protein diets (Special Diets Service) were also isoenergetic (3.9 kcal AFE/g). Mice were weighed on a regular basis to assess growth rate.

### Genotyping of Mice by PCR

Ear (UK) or tail (USA) biopsies were taken from mice either aged ≥21 days or from pups sacrificed on date of birth. Genomic DNA was extracted from the biopsies using the DNeasy Blood and Tissue kit (Qiagen) or the microLysis-plus solution (Web Scientific). To determine the genotypes of individual mice, three sets of primers were used (see Figure 1, Table 1). For each genotyping reaction, 2 µl of genomic DNA was used with 1 µM of each primer and the GoTaq Green Master Mix (Promega). For 9–10 and 9–13 sets of primers, the PCR program was 95°C, 3 min; [95°C, 30 s; 61°C, 30 s; 72°C, 1 min] (40 cycles); 72°C, 2 min. And for the Cre set of primers (18–19), the PCR program was 94°C, 4 min; [94°C, 1 min; 55°C, 2 min; 72°C, 3 min] (35 cycles). PCRs were performed using a G-Storm GS1 thermal cycler and PCR products were resolved on 2% (w/v) agarose gels containing SYBR safe DNA stain (Invitrogen) in TAE buffer and imaged under UV light.

### Glucose Tolerance Test

After an overnight fasting (8 h), initial blood glucose level was monitored from tail vein blood using the AlphaTRAK Blood Glucose Monitoring System (Abbott). Then, glucose (2 mg/g body mass) was administered intraperitoneally and blood glucose monitored by tail bleeding over a 2 h period.

### Leucine Injection

After an overnight fasting (8 h), NaCl (0.9%, w/v) or various doses of Leucine (5, 10, 20, 40, 100, 200 µg/g body mass) were administered intraperitoneally. Mice were sacrificed and dissected 10 min after injection.

### Blood and Tissue Collection

Mice were killed by CO_2_ inhalation, blood was collected by cardiac puncture and tissues were rapidly collected and weighed; the intestines were flushed with PBS. Tissues were immediately frozen in liquid N_2_ and stored at −80°C until processing. The blood was centrifuged (1000 g, 20 min) in heparinized tubes to separate into plasma and cells. Plasma insulin levels were determined using a commercial ELISA kit (Mercodia).

### RNA Extraction from Mouse Tissues and cDNA Synthesis

Harvested mouse tissues were ground to a powder under liquid N_2_ with a mortar and pestle, ≤30 mg of tissue were lysed using the TRI Reagent (Sigma-Aldrich) and RNA was extracted using the RNeasy mini kit (Qiagen) according to the manufacturer’s instructions. Samples were DNase treated ‘on-column’ using RNase-free DNase (Qiagen) according to the manufacturer’s instructions. RNA concentration was determined using a Nanodrop (Agilent Technologies) and 500 ng was used to synthesize single strand cDNA in 20 µl reactions using the qScript cDNA Synthesis Kit (Quanta Biosciences), the resultant cDNA was stored at −20°C until use.

### Quantitative Real Time Reverse-transcriptase PCR

Quantitative PCR (qPCR) primers were designed using the NCBI primer blast tool (http://www.ncbi.nlm.nih.gov/tools/primer-blast/) to produce amplicons crossing an exon boundary; specific sequences are reported in Table 2. In the generated mouse line described herein, exon1 of the *Slc7a5* gene is targeted for Cre-LoxP mediated excision, so primer sets were designed spanning the 1–2 exon boundary of the *Slc7a5* gene to test for selective knockout of *Slc7a5* in mice of differing *Slc7a5* transgenic genotypes. For each qPCR, Slc7a5 was normalised to β-Actin concentration using gene specific primers. Single strand cDNA synthesized from mouse tissue RNA was diluted 1∶5 (intestine), 1∶10 (heart) or 1∶2 (diaphragm, gastrocnemius) with nuclease free water and qPCR was performed in a 96-well format using an Applied Biosystems StepOne thermal cycler (Applied Biosystems Life Technologies). Reactions consisted of 2 µl of diluted cDNA per well, SYBR Green JumpStart *Taq* ReadyMix (Sigma-Aldrich) and 0.5 or 1 µM of each primer in a 20 µl total reaction. Thermal cycling conditions were an initial denaturation step of 95°C for 15 mins, and then 40 cycles of 94°C for 15 secs, 56°C for 30 secs and 72°C for 30 secs; stasis at 4°C until analysis. Each experimental cDNA was measured in triplicate with both Slc7a5 and β-Actin primers. For each plate, a standard curve was produced using both Slc7a5 and β-Actin primers and sequential dilutions (5×1∶5) of a cDNA synthesized from control mouse RNA (Zygene). The relative quantity of Slc7a5 mRNA in each sample was normalized to β-Actin mRNA abundance using StepOne software and qPCR results expressed as (Slc7a5/β-Actin) mRNA.

**Table 2:** Sequences of primers used to quantify mRNA by PCR (Q-PCR analysis) alongside the melting temperature (Tm) of each primer set.

Gene	Sequence 5′ to 3′	Length (bp)	% GC content	Tm (°C)	Product length (bp)
β-Actin forward	ATGCTCCCCGGGCTGTAT	18	61	60.5	63
β-Actin reverse	CATAGGAGTCCTTCTGACCCATTC	24	50	60.2	
Slc7a5 forward	CTGGTCTTCGCCACCTACTT	20	55	59.4	127
Slc7a5 reverse	GCCTTTACGCTGTAGCAGTTC	21	52	59.6	
Slc7a8 forward	AAGAAGCCTGACATTCCCCG	20	55	60.0	180
Slc7a8 reverse	TGTGTTGCCAGTAGACACCC	20	55	59.9	
ATF-4 forward	AGCAAAACAAGACAGCAGCC	20	50	59.6	192
ATF-4 reverse	ACTCTCTTCTTCCCCCTTGC	20	55	59.0	

### Immunoblot

To provide good negative and positive controls, mice were intraperitoneally injected with NaCl (0.9% w/v) or Insulin (2 mU/g body mass) after a 6 h fasting period. Tissues were collected after 10 min. Heart, gastrocnemius (50 mg) or soleus muscles (12 mg) were homogenized using a Polytron in lysis buffer [50 mM Tris/HCl (pH 7.4), 0.27 M sucrose, 1 mM sodium orthovanodate, 1 mM EDTA, 1 mM EGTA, 10 mM sodium β-glycerophosphate, 50 mM NaF, 5 mM sodium pyrophosphate, 1% (v/v) Triton X-100, 0.1% (v/v) 2-mercaptoethanol and Protease Inhibitors (Roche)]. Lysates (60 µg) were separated by SDS-PAGE, and transferred to Immobilon-P membranes (Fisher Scientific). Blots were probed with antibodies recognizing phospho-S6K [Thr389], S6K, phospho-AMPK [Thr172] (1∶1000 dilution) (Cell Signaling Technology), AMPKα1/2 (purified sheep polyclonal antibody to a peptide TSPPDSFLDDHHLTR for α1 and MDDSAMHIPPGLKPH for α2–1∶5000 dilution) [18], actin (1∶2000 dilution) (Sigma Aldrich), SLC7A5 (purified rabbit polyclonal antibody to a peptide CQKLMQVVPQET) (1∶250 dilution) [19]. Primary antibody detection was performed with the appropriate HRP (horseradish peroxidase)-conjugated anti-rabbit or anti-mouse IgG and resulting signals visualized using enhanced chemiluminescence by exposure to Amersham hyperfilm ECL (GE Healthcare). For phospho-AMPK [Thr172] and AMPKα1/2 antibodies, detection was performed using secondary antibody (1 µg/ml) coupled to IR 680 or IR 800 dye, and the membranes scanned using the Li-Cor Odyssey IR Imager. Immunoblots were quantified using the ImageJ software.

### Amino Acid Analysis by HPLC

20 mg of ground gastrocnemius muscle or 50 µl of plasma were homogenized in Trifluoroacetic acid and Methanol (1∶10). Supernatants were dried off in a rotary evaporator at 46°C. Samples were suspended successively in Sodium acetate, Methanol, TEA (2∶2:1), Methanol, H2O, TEA, PITC (7∶1:1∶1) and Methanol (100%) with drying steps between each step. The resulting phenylthiocarbamyl peptides were separated by a Hewlett Packard 1050 HPLC system (Minnesota, USA) with post-column UV detection (254 nm). HPLC traces were analyzed using the Clarity Lite software.

### Phenylalanine Uptake

Paired soleus muscles and hemidiaphragms were dissected from individual animals and, for each muscle type, one of the pair was incubated in Transport Buffer [121 mM NaCl, 4.9 mM KCl, 2.5 mM MgSO_4_, 20 mM Tris-HCl, 1 mM CaCl_2_, pH 7.4] containing 5 µM Phe while the other was incubated in identical buffer containing excess (20 mM) Phe. Both solutions contained ^3^H-Phe at 18.5kBq/ml. Following a timed incubation (10–15 min), the muscle samples were rapidly rinsed in PBS and lysed overnight in lysis buffer [50 mM NaOH, 1% SDS] at 55°C. The radioactivity in each sample was measured using liquid scintillation counting. Protein concentration of each sample was measured using the BCA protein assay (Pierce). The rate of saturable phenylalanine uptake into diaphragm and soleus was then calculated as (total AA uptake [^3^H-Phe tracer uptake in the presence of 5 µM Phe]) – (non-saturable AA uptake [^3^H-Phe tracer uptake in the presence of excess (20 mM) Phe]). Data is expressed as saturable uptake/mg total protein/min.

### Statistical Analysis

Results are expressed as mean value ± S.E.M for *n* measurements or experiments denoted in figure legends. Statistical analysis was performed using either two-way ANOVA (diet × genotype interactions) with Bonferroni post *t-*test or by unpaired Student’s *t-*test as appropriate using GraphPad Prism software. Significance was assigned at *p<0.05, **p<0.01, ***p<0.001.

## Results

### Generation of *Slc7a5*-Flox Mouse Line

The *Slc7a5* targeting construct (Figure 1A), which included FLPe sites for *in vivo* removal of the neomycin-IRE-SD selection domains [20], [21], was transfected into mouse ES cells. Cells with homologous recombination of the *Slc7a5* targeting construct (in which one copy of the endogenous *Slc7a5* gene from −5787 to +2875 was replaced by the corresponding regions of the targeting vector; *Slc7a5*-Flox^Neo^) were selected by neomycin-resistance and confirmed by RT-PCR using primers 11 and 14 (Figure 1B) followed by cloning and sequencing of the PCR product. Subsequently, positive cells were injected into isolated mouse blastocysts which were then implanted into the uterus of pseudopregnant female mice. Male chimeric offspring (identified by coat colour) were mated with wild-type female C57Bl/6 mice. Offspring of these mice were genotyped by PCR with primer sets 9–10 and 12–13, which allowed the identification of those animals harbouring the 5′ and 3′ loxP sites, respectively, at the desired locations (Figure 1C). Founder *Slc7a5*-Flox^Neo^ mice were crossed with FLPe recombinase-expressing mice to produce offspring in which the neomycin-IRES-SD selection cassette was removed by recombination at the FRT sites. This was confirmed by PCR using primers 15 and 13, which resulted in a 673 bp product for wild-type allele (data not shown). The FLPe transgene was bred out by backcross with C57Bl/6 mice to produce the final *Slc7a5*-Flox (FLPe-) genotype suitable for breeding with Cre-expressing mice to produce *Slc7a5* knockout offspring. Multiple self-crosses showed that heterozygous and homozygous mice with *Slc7a5*-Flox genotype (*Slc7a5* ^fl/+^ *Slc7a5* ^fl/fl^ respectively) were viable and fertile with no detectable abnormalities.

### Global *Slc7a5* Knockout is Embryonic Lethal

*Slc7a5*-Flox mice were crossed with two different strains of germline Cre recombinase-expressing mice (*Bal1*-promoter Cre and *Elalpha*-promoter Cre) to remove the Floxed exon1 of the *Slc7a5* gene. Genotyping of the knockout animals was carried out by PCR analysis using the 9–13 primer pair, which generates a product of 253 bp only with the recombined *Slc7a5* gene lacking the 1855 bp floxed region including exon1 (Figure 1D). Heterozygous *Slc7a5* gene knockout mice (both *Bal1*-Cre *Slc7a5*+/− and *Elalpha*-Cre *Slc7a5*+/−) were found to be viable, fertile and phenotypically similar to wild-type littermates with equivalent growth profiles and food intakes (data not shown). Numerous discrete self-crosses of *Slc7a5*+/− mice failed to produce offspring with a genotype of *Slc7a5*−/− for either Cre line (p<0.01; Χ^2^-test based on expected Mendelian frequencies, N = 108 live births). A global homozygous *Slc7a5*-knockout therefore appears to be embryonic lethal. The *Bal1*-Cre and *Elalpha*-Cre transgenes were bred out by backcross with C57Bl/6 mice to produce *Slc7a5*+/− mice for physiological studies.

### Functional Reduction in Slc7a5 Expression in *Slc7a5*+/− Mice

**Figure 2:**
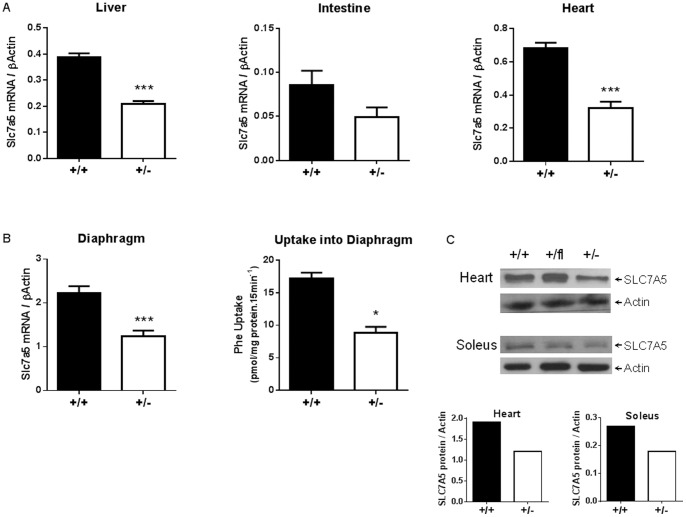
Both *Slc7a5* gene expression and SLC7A5 transport activity are reduced in *Slc7a5*+/− mouse tissues. (A) Slc7a5 mRNA expression in liver, heart and intestine (as indicated) from *Elalpha*-Cre *Slc7a5*+/+ (n = 5–9) and *Slc7a5*+/− (n = 4–5) mice as determined by qPCR and normalized to β-Actin. Intestine was determined not significant (N.S.) although p<0.1. (B) Slc7a5 mRNA expression in diaphragm from *Slc7a5*+/+ and *Slc7a5*+/− mice as determined by qPCR and normalized to β-Actin (n = 6). Uptake of ^3^H-phenylalanine into diaphragm from *Slc7a5*+/+ and *Slc7a5*+/− mice (n = 5). *and ***indicate p<0.05 and p<0.001 respectively by unpaired t-test. (C) Representative Western blot of SLC7A5 protein in heart and soleus muscle lysates from from *Slc7a5*+/+ and *Slc7a5*+/− mice. Blot quantitation shown in lower panels.

Slc7a5 mRNA expression was significantly reduced (by around half) in heart, skeletal muscle (*e.g.* diaphragm) and liver of *Slc7a5*+/− mice in comparison with *Slc7a5*+/+ littermates (Figure 2). Reduced SLC7A5 protein expression was also detected in heart and soleus muscle of *Slc7a5*+/− mice relative to *Slc7a5*+/+ littermates (Figure 2C). We confirmed a reduction in functional SLC7A5 expression in diaphragm muscle by demonstrating that 5 µM phenylalanine uptake was significantly lower in *Slc7a5*+/− diaphragm compared to wild-type controls (Figure 2B). Nevertheless, there were no significant differences between mass of tissues including skeletal muscles (gastrocnemius, soleus), heart or liver for *Slc7a5*+/− mice and wild-type littermates (data not shown), nor in AA content of tissues or plasma (Table S1).

### Reduced Functional Slc7a5 Expression in Muscle-specific MCK-Cre *Slc7a5^fl/fl^* (MS-*Slc7a5*-KO) Mice

**Figure 3:**
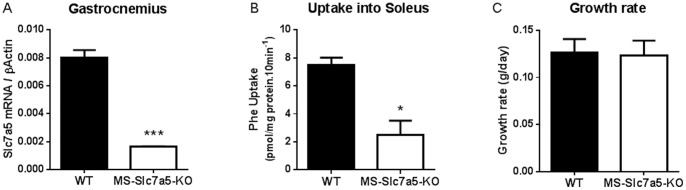
*Slc7a5* gene expression and SLC7A5 transport activity in MS-*Slc7a5*-KO mouse muscles and impact on tissue growth. (A) Reduced expression of Slc7a5 mRNA in MS-*Slc7a5*-KO mouse gastrocnemius muscle (n = 24) compared to wild-type (n = 36). ***indicates p<0.001 by unpaired t-test. (B) Reduced SLC7A5 (System L1) transport function (measured as Phe uptake) in MS-*Slc7a5*-KO soleus muscle (n = 4) compared to wild-type (n = 6) *indicates p<0.05 by unpaired t-test. (C) No difference on the growth rate of MS-*Slc7a5*-KO and wild-type mice on standard chow diet (14% protein) (n = 5).

**Figure 4:**
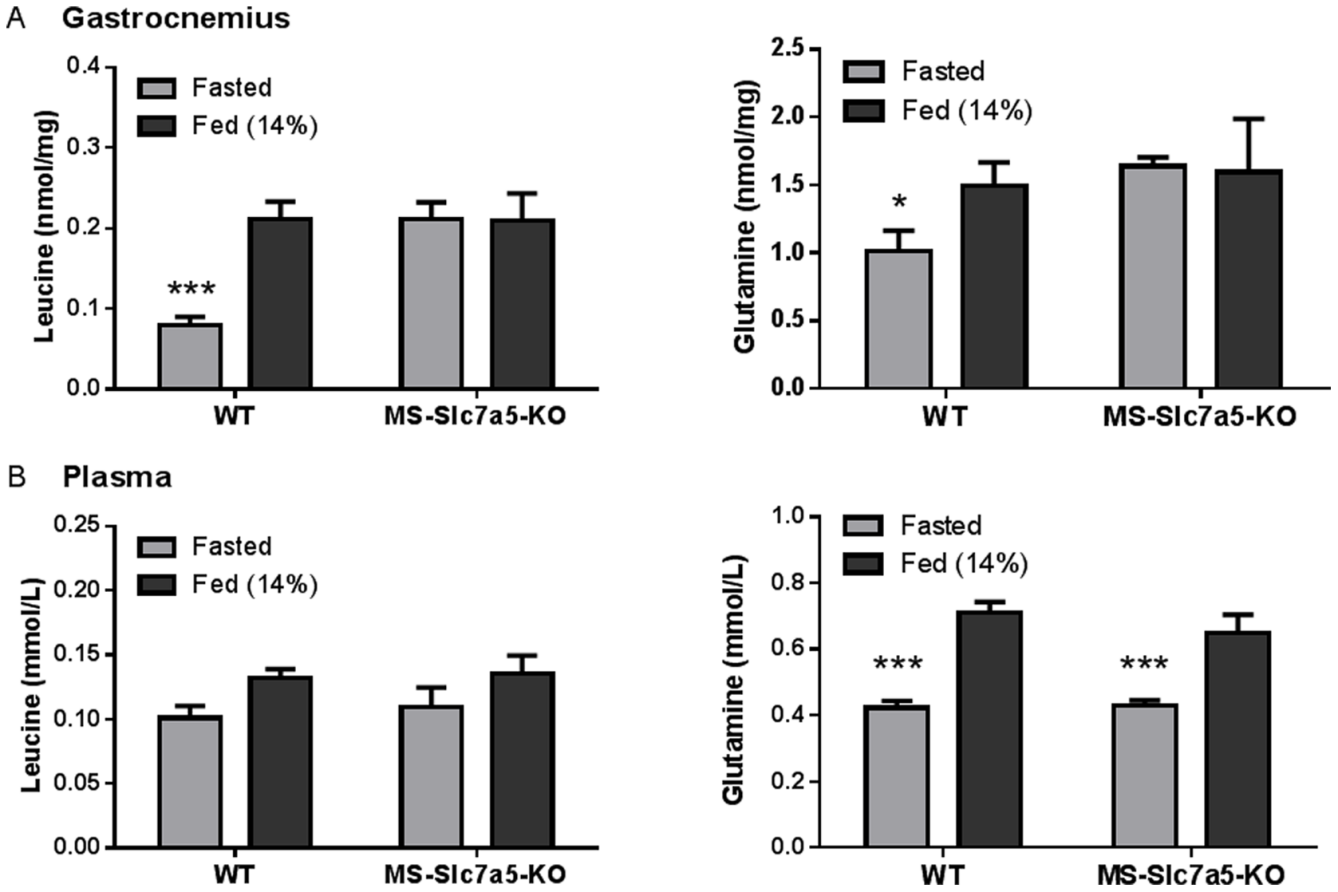
Effect of 8 h overnight fast on muscle and plasma amino acid (leucine, glutamine) concentrations in MS-*Slc7a5*-KO mice. Mean ± SEM for n = 6–9 (WT) and 3–5 (MS-*Slc7a5*-KO) mice. (A) Intramuscular AA are significantly lower after 8 h fast in WT animals but not MS-*Slc7a5*-KO animals. *and ***indicate p<0.05 and p<0.001 respectively by Bonferroni’s post *t-*test). (B) Plasma glutamine concentrations are significantly lower after 8 h fast for both genotypes (***indicates p<0.001 by Bonferroni’s post *t-*test), although an overall effect of fasting on plasma leucine is detected (*F* (1, 19) = 5.49, *p* = .030), the difference does not achieve p<0.05 for either genotype.

In the absence of viable *Slc7a5*−/− progeny, we were unable to investigate the global importance of SLC7A5 to nutrient-signalling upstream of the mTORC1 pathway and its functional consequences. Given the important protein-anabolic effects of mTORC1 activation, we decided to focus on the effects of *Slc7a5* knockout in skeletal muscle, the tissue with greatest protein mass in the body. *Slc7a5^fl/fl^* mice were crossed with mice expressing Cre recombinase downstream of a muscle-specific promoter, MCK (muscle creatine kinase promoter) – Cre [22], to produce a muscle-specific (MCK-Cre) knockout of *Slc7a5.* MCK-Cre *Slc7a5^fl/fl^* (MS-*Slc7a5*-KO) mice were viable, fertile and born at standard Mendelian frequency. Genotyping PCR of gastrocnemius muscle confirmed Cre-dependent *Slc7a5* gene excision (Figure S1B) and analysis of *Slc7a5* gene expression in gastrocnemius muscle by qPCR confirmed a substantial reduction of Slc7a5 mRNA expression in muscle of MS-Slc7a5-KO mice (Figure 3). The residual Slc7a5 mRNA in whole muscle extract is likely to be largely expressed in reticuloendothelial and fibrous tissue [7], [23]. Soleus and diaphragm muscles from these mice both showed significant reductions in phenylalanine uptake consistent with functional knockout of the *Slc7a5* gene (Figures 3B and S1A), although there was no overt growth phenotype for MS-Slc7a5-KO mice (Figure 3C). There were no significant differences in intramuscular or plasma concentrations of leucine or glutamine (two key SLC7A5 substrates) between MS-Slc7a5-KO and control mice fed *ad libitum* on laboratory chow diet (14% protein) (Figure 4). In contrast, fasting (8 h over dark period) produced significant reductions in intramuscular concentrations of leucine and glutamine only in wild-type animals (Figure 4), indicating that these AA may be less able to efflux from skeletal muscles of MS-*Slc7a5*-KO mice; similar effects were noted for other neutral AA (Figure S2A). Plasma AA concentrations of both mouse genotypes showed similar changes with fasting (Figures 4B and S2).

### Reduced Leucine-activated mTORC1 Signalling in Skeletal Muscle of MS-*Slc7a5*-KO Mice

**Figure 5:**
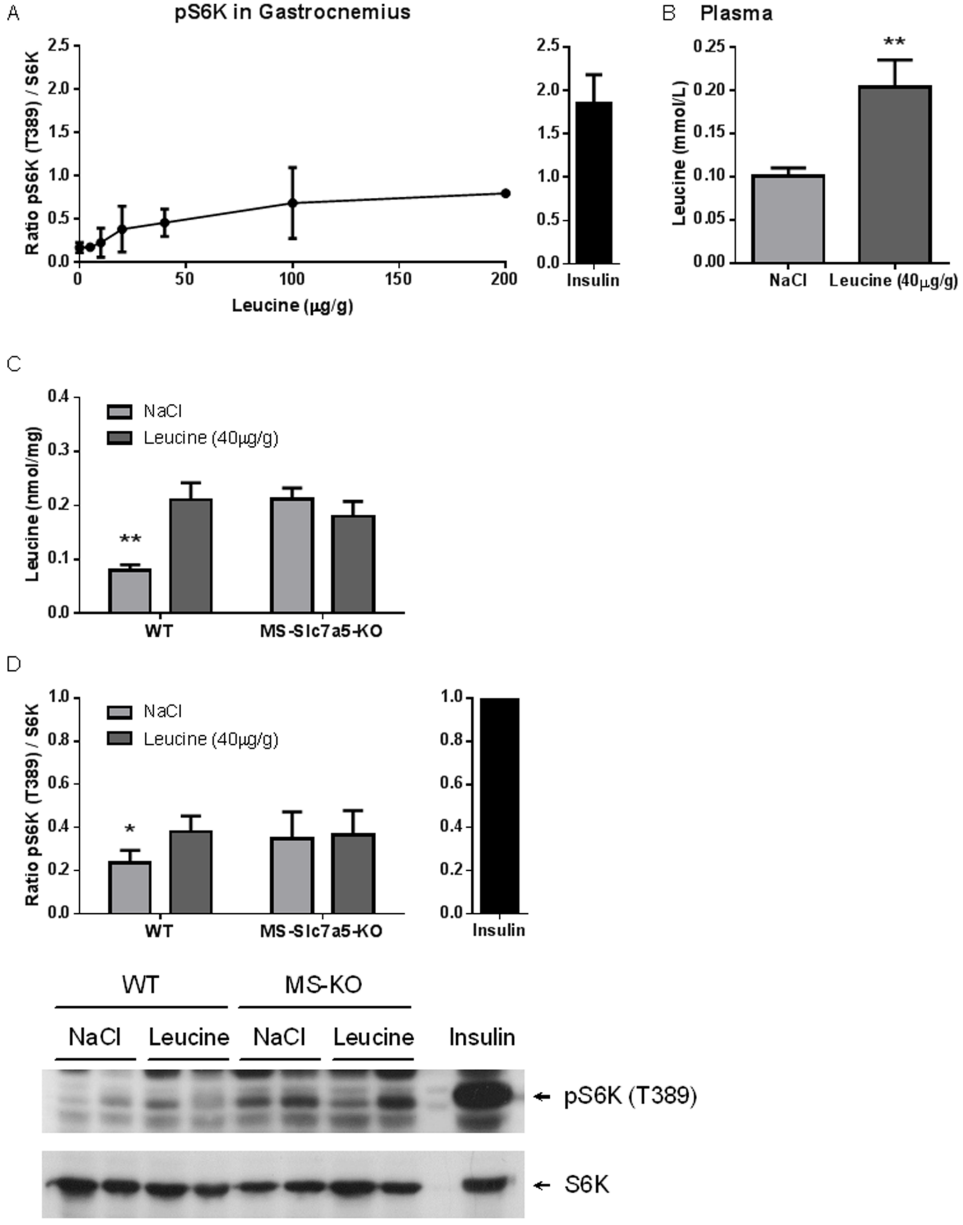
Intraperitoneal leucine injection activates mTORC1 signalling in skeletal muscle. (A) Concentration-dependent effect of I.P. leucine injection on mTORC1-S6K pathway activation in gastrocnemius muscle from fasted wild-type mice, compared to effect of insulin injection (sampling 10 minutes post-injection in all cases) as shown by ratio of pS6K (T389)/S6K. (B) Effect of 40 µg/g leucine injection on plasma leucine concentration at time of tissue sampling (n = 9–10, **indicates p<0.01). (C), (D) Effect of 40 µg/g leucine injection on (C) intramuscular leucine concentration and (D) Leu-induced mTORC1-S6K pathway activation at time of tissue sampling (10 min post-injection) in gastrocnemius muscle from fasted wild-type and MS-*Slc7a5*-KO mice. Mean ± SEM for n = 8–12 (WT) and 5–6 (MS-*Slc7a5*-KO) mice. Lower panel in (D) shows a representative phospho-S6K blot. Both S6K phosphorylation and muscle leucine concentration are significantly increased following leucine injection in wild-type (*and **indicate p<0.05 and p<0.01 respectively) but not MS-*Slc7a5*-KO animals. Activation of S6K by leucine (*i.e.* the difference between T389 phosphorylation in muscle from leucine - injected and vehicle (NaCl) – injected mice) appears to be blunted in MS-*Slc7a5*-KO muscle, Animals injected with insulin were used as a positive control in (B) and the value of pS6K/S6K was set to 1 for the insulin injected animals.

We next investigated the ability of leucine to activate mTORC1 signalling *in vivo* by intraperitoneal injection of leucine in 8-hour fasted wild-type mice. 10 minutes post-injection, we observed a dose-dependent activation of muscle S6K (a downstream mTORC1 target) at the rapamycin-sensitive Thr389 phosphorylation site (Figure 5A). The maximum activation of S6K by leucine was less than 50% of the effect achieved when mice were injected with insulin. We chose a leucine dose giving robust but submaximal responses (40 µg/g) for further study, which resulted in an approximate doubling of both plasma and intramuscular leucine concentrations at the 10 minutes sampling point in wild-type mice (Figures 5B, C), illustrating rapid replenishment of the muscle leucine pool depleted by fasting. In contrast, there was no increase in intramuscular leucine concentration after leucine injection in MS-*Slc7a5*-KO mice (Figure 5C), although the initial fasting intramuscular leucine concentration was higher than for wild-type mice. There was a higher basal Thr389 phosphorylation of S6K in MS-Slc7a5-KO mice compared to wild-type (Figure 5D), but this remained submaximal for leucine-induced activation (*c.f*. Figure 5A). The activation of S6K by injected leucine appeared to be blunted in muscles of MS-*Slc7a5*-KO mice (Figure 5D).

### Effect of Altered Dietary Protein Intake on Tissue AA Concentrations and mTORC1 Signalling in Skeletal Muscle of MS-*Slc7a5*-KO Mice

**Figure 6:**
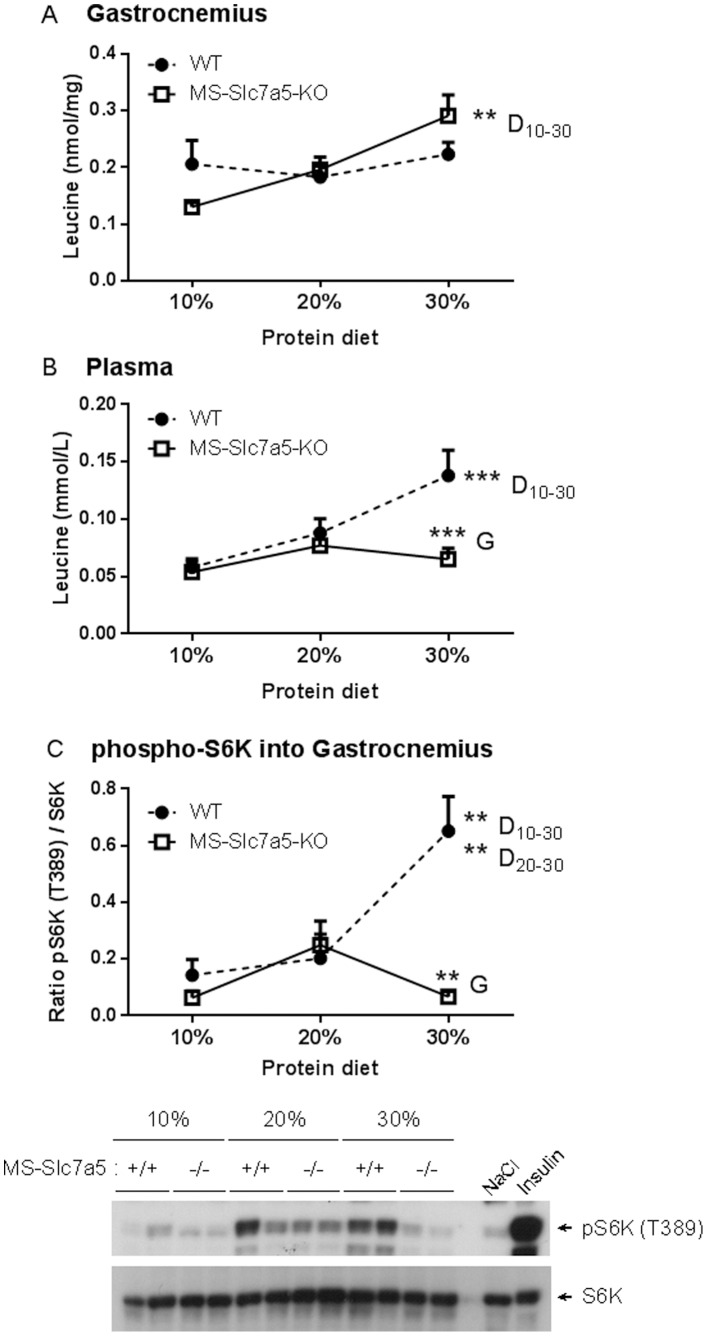
Altered dietary protein intake affects plasma and intramuscular AA concentrations and mTORC1 pathway signalling in MS-*Slc7a5*-KO mice. Mean ± SEM for n = 6–10 (WT) and 3–5 (MS-*Slc7a5*-KO) male mice. (A) There were significant effects of dietary protein content (*F* (2, 27) = 4.97, *p* = .014) on gastrocnemius leucine concentration. Statistically-significant differences between groups were only detected for MS-*Slc7a5*-KO animals on different protein diets (**D, p<0.01) as indicated. (B) There were significant effects of both genotype (*F* (1, 23) = 5.55, *p* = .027) and dietary protein content (*F* (2, 23) = 4.49, *p* = .023) on plasma leucine concentration. Statistically-significant differences between genotype (***G, p<0.001) and dietary protein (***D, p<0.001; wild-type only) groups are indicated. (C) Upper panel shows quantitation of S6K phosphorylation normalised to effect of insulin injection (wild-type mouse on standard chow diet), lower panel shows representative western blot for phospho-S6K and total S6K. Animals injected with NaCl and insulin were used as a negative and positive control, respectively. 2-way ANOVA shows significant effects of both genotype (*F* (1, 27) = 9.61, *p* = .004) and dietary protein content (*F* (2, 27) = 4.97, *p* = .015) on S6K phosphorylation. Statistically-significant differences between genotype (**G, p<0.01) and diet (**D, p<0.001; wild-type only) groups are indicated.

In order to provide further insight into possible impairment of nutrient-signalling by muscle-specific *Slc7a5* knockout, MS-*Slc7a5*-KO and control mice were challenged with diets of 10%, 20% or 30% protein (low, control and high protein diets respectively) from age 40 days for a 60-day period. Dietary protein compositions were chosen to maintain equivalent energy intake by minimizing possible effects on food intake. No difference was observed in growth rates or food intake between genotypes or diets even though a trend for small (<5%) reductions in heart and skeletal muscle size was noted for MS-*Slc7a5*-KO mice on the different diets (Figure S3). In wild-type mice, the leucine concentration in gastrocnemius muscle was maintained at around 0.2 nmol/mg across the 10–30% dietary protein range, despite a progressive increase in plasma leucine concentration. In contrast, intramuscular leucine concentration in MS-*Slc7a5*-KO animals increased in relation to dietary protein content whereas plasma leucine concentration remained unaltered (Figures 6A and B). A similar pattern was observed for isoleucine (Figure S4). On the 30% diet, leucine, isoleucine and glutamine accumulated in muscle of MS-*Slc7a5*-KO mice relative wild-type mice (Figures 6 and S4). In non-fasted wild-type mice, intramuscular (gastrocnemius) mTOR-S6K activity increased with dietary protein intake (Figures 6 and S5) and was consistently higher than mTOR-S6K activity in muscle from MS-*Slc7a5*-KO mice on both high- and low-protein diets (Figures 6C and S5). This was observed in gastrocnemius muscle of both male and female mice and also in soleus muscle (Figures S5A and S5B respectively).

### Effect of Altered Dietary Protein Intake on Indices of Insulin-sensitivity in Skeletal Muscle of MS-*Slc7a5*-KO Mice

**Figure 7:**
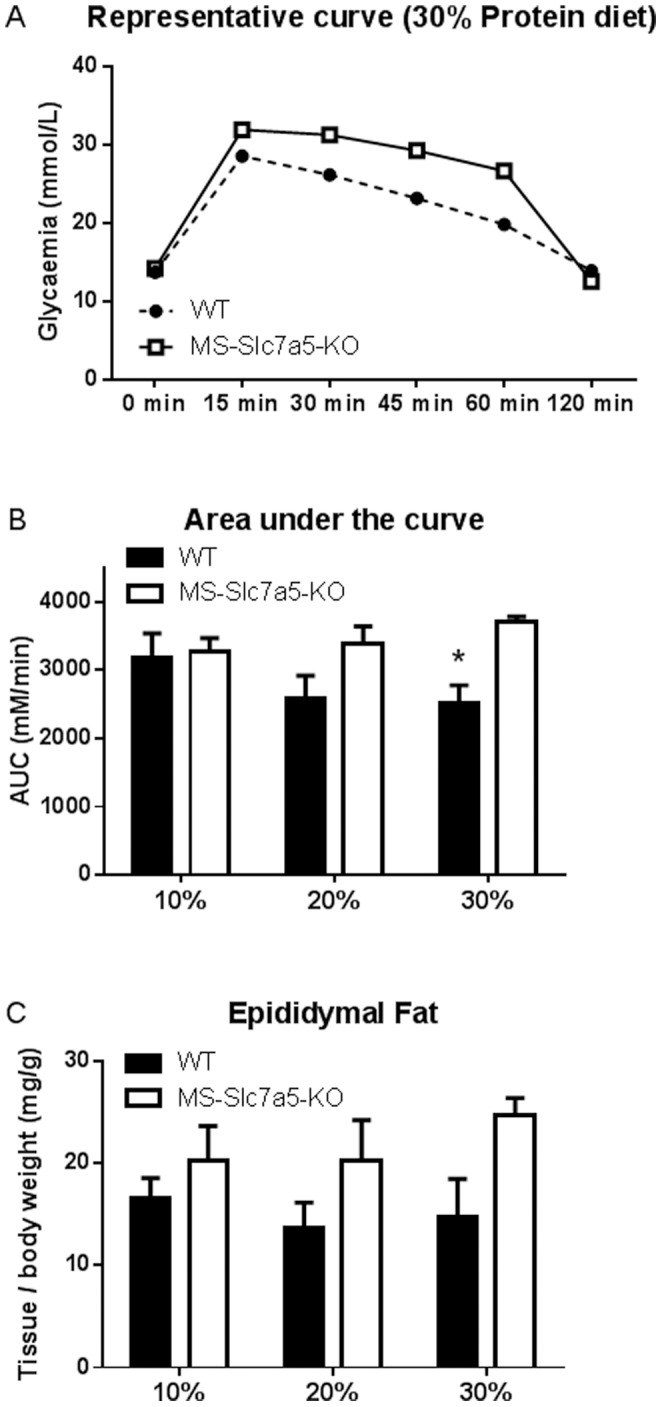
Altered dietary protein intake influences indices of insulin-sensitivity in MS-*Slc7a5*-KO mice. (A) Representative results of blood glucose concentration (mmol/L) in mice after intraperitoneal glucose injection during glucose tolerance test (GTT) on mice fed a 30% protein diet. (B) Quantitation of GTT responses presented as area-under-curve (AUC) for glucose disposal. Mean ± SEM for n = 5–7 (WT) and 4–5 (MS-*Slc7a5*-KO) male mice: 2-way ANOVA shows significant effects of genotype (*F* (1, 24) = 8.62, *p* = .007) on AUC: Statistically-significant differences between genotype (*, p<0.05) are indicated. (C) Gonadal epididymal fat mass of male mice at time of tissue sampling. Mean ± SEM for n = 6–7 (WT) and 4–5 (MS-*Slc7a5*-KO) mice. 2-way ANOVA reveals a significant effect of genotype (*F* (1, 27) = 6.54, *p* = .016) on gonadal fat mass, but the difference does not achieve p<0.05 at any specific protein intake.

In view of the reduced mTORC1 pathway activation in muscle of MS-*Slc7a5*-KO mice challenged with high- or low-protein diets and the importance of mTORC1 signalling to insulin action, we evaluated insulin sensitivity of experimental mouse groups (male animals only) on different protein diets. Within the range of dietary protein studied (10–30%), we measured a graded increase in insulin sensitivity (*i.e.* improved glucose clearance) in wild-type animals from the 10% (low protein) to the 30% (high protein) diet (Figure 7). However, the MS-*Slc7a5*-KO mice showed no such increase, indicating that these animals may become relatively insulin-resistant on a high-protein diet. Furthermore, these mice tended towards a greater gonadal fat mass (Figure 7C), although plasma insulin levels were not significantly different between genotypes in the fed state (Figure S6).

### Effect of Altered Dietary Protein Intake on Activity of Other Growth and Nutrient-related Signalling Pathways in Skeletal Muscle of MS-*Slc7a5*-KO Mice

**Figure 8:**
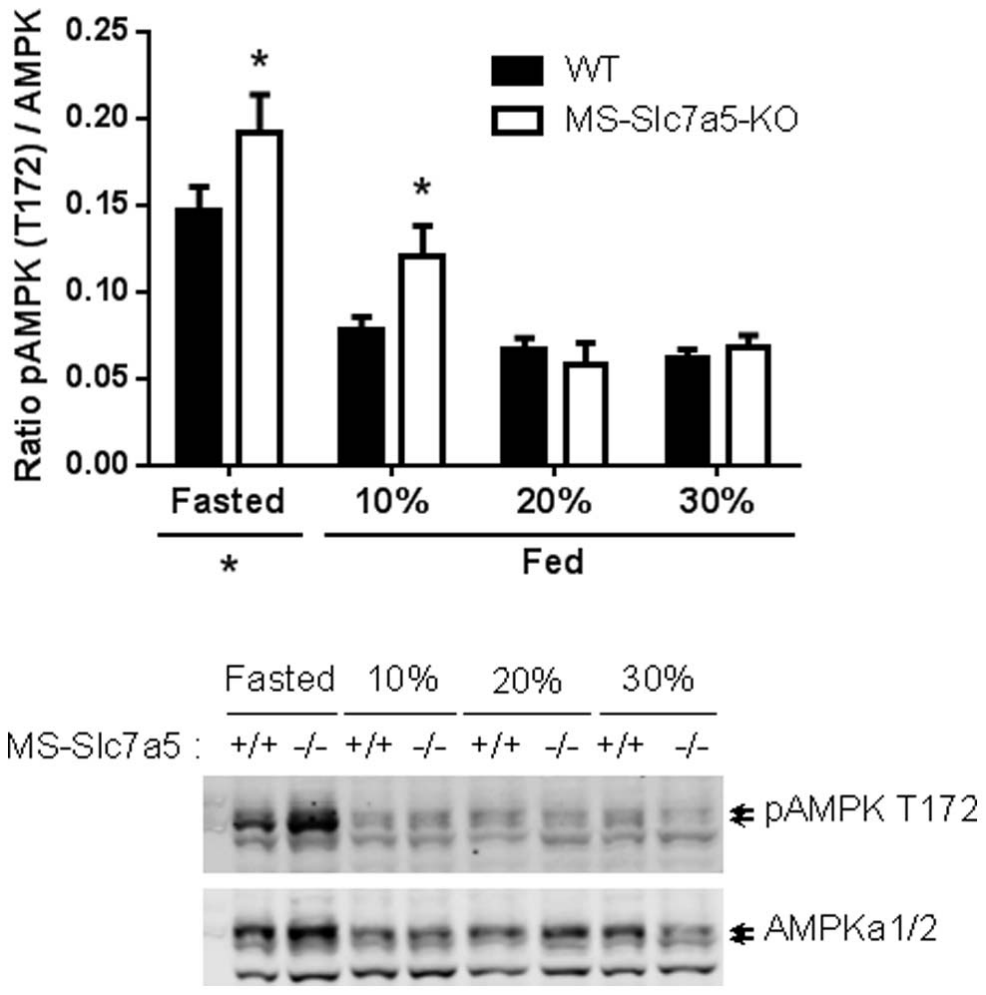
Effect of altered dietary protein intake on AMPK pathway activation in gastrocnemius muscle of MS-*Slc7a5*-KO mice. Upper panel shows quantitation of all measurements (mean ± SEM for n = 10–14 (WT) and 5–9 (MS-*Slc7a5*-KO) mice), lower panel shows representative western blot for phospho-AMPK. 2-way ANOVA shows significant effects of both genotype (*F* (1, 69) = 8.07, *p* = 0.006) and dietary status (*F* (3, 69) = 43.8, *p*<0.0001) on AMPK phosphorylation. Statistically-significant differences between genotype are indicated (*, p<0.05). Fasted values for both genotypes are significantly different from fed values, irrespective of dietary protein content.

Activation level of the AKT/PKB (Thr308 phosphorylation) growth-factor signalling pathway was not affected by *Slc7a5* genotype or dietary protein content (Figure S6C). An effect of dietary leucine supplementation in the improvement of insulin sensitivity has been linked to action of the energy-sensitive AMPK (5′ adenosine monophosphate-activated protein kinase) pathway [14]. Activation of the cellular-energy sensor AMP kinase leads to an inhibition of mTORC1 [24] and may override activation by leucine when energy-status is low [25]. AMPK activation was significantly higher in fasted mouse gastrocnemius muscle compared to the fed state for both genotypes (Figure 8). There was a significant increase in intramuscular AMPK activation in MS-*Slc7a5*-KO compared to wild-type in both fasted and 10% protein-fed mice (Figure 8), perhaps indicative of an increase in metabolic stress under protein-scarce conditions. However, the expression of Atf4 mRNA in gastrocnemius muscle, a more specific indicator of low AA stress, was not affected either by genotype or diet (Figure S7).

## Discussion

We show that global knockout of *Slc7a5* by Cre-mediated excision of a region including exon1 from the *Slc7a5* gene results in an embryonic lethal phenotype in mice, although the precise timing and cause are still under investigation. Mice lacking the domain within SLC3A2 (CD98hc) that is required for interaction with SLC7 light-chain permeases such as SLC7A5 also do not survive early embryogenesis [26], which may be partly due to a deleterious effect upon SLC7A5 transport function during post-implantation murine embryonic development [27]. No *Slc7a5* mutations associated with inherited human diseases have been reported (perhaps unsurprising given that the gene appears to be essential for mammalian embryonic development), although some other members of the SLC7 family are linked with genetic disorders [7], [28]. *Slc7a5*+/− mice display the anticipated ∼50% reduction in *Slc7a5* gene expression but otherwise appear to have a normal anatomical and physiological phenotype. *Slc7a5* gene deletion is unlikely to affect intestinal absorption or renal reabsorption of LNAA because of the relatively low *Slc7a5* expression in epithelial tissues [7].

Muscle-specific knockout of the *Slc7a5* gene (MS-*Slc7a5*-KO) in mice results in substantial reductions of both Slc7a5 mRNA expression and LNAA transport function in the skeletal muscles studied. Skeletal muscle expresses both high-affinity and low-affinity System L1 transporters for LNAA (SLC7A5 and SLC7A8 respectively), in common with several other tissues [7]. The low phenylalanine concentration (5 µM) used for our *in vitro* transport studies disproportionately favours uptake by the high-affinity SLC7A5 transporter over other LNAA transporters expressed in muscle, so the reduction of muscle LNAA uptake *in vivo* due to *Slc7a5* knockout is likely to be less than the ∼75% reductions shown in Figures 2B, 3B and S1A. SLC7A8 mRNA is upregulated in MS-*Slc7a5*-KO mouse muscle (Figure S1C), therefore SLC7A8 may provide partial functional compensation of the LNAA transport deficit due to absence of SLC7A5. Nevertheless, our results (assuming that they are reflective of changes in all skeletal muscles) imply that normal muscle size may be maintained in adult mice with a significantly diminished LNAA influx, given that the gastrocnemius muscles of MS-*Slc7a5*-KO mice are not significantly smaller than in wild-type animals. Consistent with this viewpoint, the *Slc7a8* (LAT2) knockout mouse has no overt metabolic phenotype except for neutral aminoaciduria [29].

Phenotypic differences between MS-*Slc7a5*-KO and wild-type animals were only revealed when mice were nutritionally challenged, either by fasting or maintenance on high/low protein diets. MS-*Slc7a5*-KO mice do not maintain the intramuscular leucine homeostasis achieved by wild-type mice across the 10–30% dietary protein range. When extracellular leucine availability is chronically restricted (*e.g.* on 10% protein diet), MS-*Slc7a5*-KO muscles appear unable to maintain normal concentrations of SLC7A5 substrates such as leucine and isoleucine. LNAA uptake from the plasma through SLC7A5 may therefore become limiting as a source for the intramuscular LNAA pool when dietary protein is scarce. In contrast, both leucine and glutamine show accumulation in MS-*Slc7a5*-KO skeletal muscle relative to wild-type on the 30% diet and acutely in the fasted state. These latter observations hint at a perhaps surprising additional role for SLC7A5 as a pathway for net leucine and glutamine efflux from muscle in postabsorptive stages of the dietary cycle. A dual role for SLC7A5 in both taking up and releasing LNAA across the muscle plasma membrane, depending upon dietary status, is consistent with the reversible exchange mechanism of operation of this transporter. The reduced ability of MS-*Slc7a5*-KO muscle to release glutamine does not significantly impact on plasma glutamine concentration in our dietary studies, consistent with the conclusion that muscle glutamine synthesis is dispensable in fed mice [30]. The increased intramuscular glutamine accumulation in MS-*Slc7a5*-KO muscle on the high-protein diet may reflect increased ammonia production from oxidation of other amino acids. The increased retention of SLC7A5 substrates (LNAA such as leucine and isoleucine) within MS-*Slc7a5*-KO muscles seen during fasting and on the high-protein diet is accompanied by elevated intramuscular concentrations of Small Neutral AA (SNAA, including alanine and glycine) which are not SLC7A5 substrates. This may occur because SNAA efflux through SLC7A8 (LAT2) may be outcompeted (and/or influx *trans*-stimulated) by the intracellular accumulation of LNAA. Indeed, AA substrate affinities of both SLC7A5 and SLC7A8 are markedly asymmetrical at intracellular and extracellular membrane faces, to the extent that the intracellular AA substrate concentration would be expected to determine their activity [31]. This type of competitive effect has been suggested to account for some symptoms related to high tissue concentrations of leucine in children with branched-chain ketoacid dehydrogenase deficiency (maple syrup urine disease) [32], [33].

Leucine injection in fasted wild-type mice produces a significant increase in intramuscular leucine concentration coincident with a significant activation of the mTORC1 target S6K. The exact mechanism by which leucine activates mTORC1 signalling is still not fully resolved but both cytosolic and lysosomal leucine-sensors have been described [1], [2]. Leucine-induced activation of mTOR-S6K appears to be blunted in MS-*Slc7a5*-KO gastrocnemius muscle, which is consistent with the suggestion that availability of SLC7A5 to rapidly replenish intracellular LNAA pool(s) after AA depletion underlies one important aspect of AA signalling to mTORC1 [34]. On the other hand, we see no simple direct relationship between intracellular leucine concentration and muscle mTORC1 pathway activity for mice of either genotype when comparing animals on 10, 20 and 30% protein diets: Wild-type animals show a progressive increase in mTORC1-S6K activation at constant intramuscular leucine concentration, which contrasts with the low level of mTORC1-S6K activation despite an increased intramuscular leucine concentration in MS-*Slc7a5*-KO mice. These relationships, which are the cumulative effect of a long-term diet, differ from those seen acutely after fasting. Whilst subcellular pooling of leucine (*e.g.* into lysosomes) may partly account for differences in signalling level, the results raise questions regarding the relative importance of LNAA concentration (as would be detected by a classical receptor) and LNAA flux within muscle as monitored variables for LNAA “sensors” upstream of the mTORC1 pathway. The close correlation between mTORC1 signalling and plasma (rather than intramuscular) LNAA concentrations resembles that reported in human skeletal muscle in response to an essential AA load [35], which has been suggested to result from signalling downstream of an extracellular essential AA sensor monitoring rises in plasma AA such as leucine. Whilst there is accumulating evidence for the existence of AA transceptors in mammalian cell membranes [36], the results may also be explained in terms of a change in essential AA flux through cell-surface and intracellular sensing-signalling pathways linked to mTORC1 activation in endolysosomes. Despite substantial reductions in mTOR-S6K activation in muscles of MS-*Slc7a5*-KO mice on high- or low-protein diets, the marginal effects on muscle weight indicate that LNAA-generated anabolic signals may not be critical for maintaining normal muscle mass, at least in young adult mice. In fact inactivation of the *mTOR* gene itself in mouse muscle does not initially reduce tissue growth, although it does result in fatal structural abnormalities in adult muscle types [37]. Our results with MS-*Slc7a5*-KO mice do not fully inform on the role of SLC7A5 in early muscle growth and development because the MCK-Cre transgene only becomes activated in *differentiated* muscle tissue [22], [38].

The progressive increase in insulin sensitivity observed for wild-type mice between the 10% (low protein) diet and the 30% (high protein) diet concurs with previous studies of rodents receiving isoenergetic diets of differing protein contents (*e.g.* [39]) and those in which mice were provided leucine supplementation to a high-fat diet [14], [40], [41] in amounts insufficient to reduce food intake. Human studies have also shown that dietary supplementation with branched-chain AA (BCAA, *i.e.* leucine, isoleucine, valine) [42] or high-protein diet [43] may help counteract pre-exising insulin-resistance. MS-*Slc7a5*-KO mice appear to be slightly more insulin-resistant than controls under all dietary regimes (especially on the 30% protein diet). This suggests a link between insulin signalling and the presence of SLC7A5 in differentiated muscle tissue, perhaps related to the influence of leucine/BCAA concentration and/or flux on the underlying level of mTORC1 pathway activation. Knockout of SLC6A19 (B^0^AT, the principal LNAA transporter in absorptive epithelial cells) in mice similarly leads to dysregulated nutrient signalling in tandem with reduced insulin responsiveness and impaired body-weight control [44]. In contrast, the skeletal muscles of BCATm-KO mice [45] (which have markedly-elevated body-fluid BCAA concentrations because the principal BCAA-catabolising enzyme in muscle is inactivated) exhibit an elevated mTORC1 activation in skeletal muscle associated with increases in protein turnover and basal energy expenditure alongside an improvement in insulin-sensitivity, although without any significant alteration of lean body mass [45]. The improvement in insulin-sensitivity seen for mice on a high-fat diet when given leucine-supplementation [40], is also linked to increased basal energy expenditure. We did not measure protein turnover (synthesis/degradation) or energy expenditure of mice in the present study but, if we accept that reduced mTORC1 pathway activation reflects reduced protein turnover, then we might speculate that a concomitant reduction in basal energy expenditure could be a contributing factor in the development of insulin resistance in MS-*Slc7a5*-KO mice on a high-protein diet.

The present study indicates that functional activity of the SLC7A5 leucine transporter in skeletal muscle modulates LNAA-dependent muscle mTOR-S6K signalling in mice. MS- *Slc7a5*-KO does not compromise the maintenance of normal muscle mass in adult mice, although this may be at least partly due to functional compensation by SLC7A8. SLC7A5 may also contribute to modulation of baseline insulin-sensitivity in relation to amino acid/protein nutrition, a possibility that could be further studied by investigated of the metabolic rate of MS-*Slc7a5*-KO mice and their responses to high-fat diet. Future study of a combined MS-*Slc7a5*-KO/*Slc7a8*-KO mouse strain may also provide better insight into the relationship between leucine transport, intracellular leucine concentration and mTORC1 pathway activation in skeletal muscle.

## Supporting Information

Figure S1 (A) Reduced phenylalanine transport function (measured as Phe uptake) in MCK-Cre-*Slc7a5*+/− and -*Slc7a5*−/− mouse diaphragm muscle. Unpaired *t-*test shows *p<0.05 compared with wild-type. (B) Confirmation of Cre-mediated *Slc7a5* gene excision in gastrocnemius muscle of MS-*Slc7a5*-KO mice. Representative PCR analysis performed on gastrocnemius muscle, using the 9–13 primer pair (5′F-3′R) generates a product of 253 bp only with the recombined *Slc7a5* gene lacking the 1855 bp floxed region including exon1. PCR analysis using the 18–19 primer pair (Cre) identifies presence of the Cre recombinase gene. (C) Increased expression of *Slc7a8* (LAT2) mRNA in MS-*Slc7a5*-KO mouse gastrocnemius muscle (n = 17) compared to wild-type (n = 22). **indicates p<0.01 by unpaired *t-*test.
(TIF)

Figure S2 **Effect of 8** **h overnight fast on muscle and plasma amino acid concentrations in MS-** ***Slc7a5*** **-KO mice.** (A) Intramuscular concentrations of several neutral AA are significantly lower after 8 h fast in WT animals (n = 8) compared to MS-*Slc7a5*-KO animals (n = 5) (*and ***indicate p<0.05 and p<0.001 respectively by unpaired *t-*test) (B) Plasma AA concentrations after 8 h fast are broadly similar for wild-type and MS-*Slc7a5*-KO mice (*indicates p<0.05 for glycine only).
(TIF)

Figure S3 **Effect of altered dietary protein intake on growth rate and muscle mass in MS-** ***Slc7a5*** **-KO mice.** Mean value ± SEM for n = 6–7 (WT) and 4–5 (MS-*Slc7a5*-KO) male mice. (A) Shows the growth rate calculated between days 60 and 80 of age. (b,c) Show the ratio between organ (gastrocnemius (B) and heart (C)) and body weight. No significant effects were detected by 2-way ANOVA.
(TIF)

Figure S4 **Effect of altered dietary protein intake on muscle and plasma concentrations of isoleucine and glutamine in MS-** ***Slc7a5*** **-KO mice.** Mean ± SEM for n = 6–10 (WT) and 3–5 (MS-*Slc7a5*-KO) male mice. (A) There were significant effects of dietary protein content (*F* (2, 27) = 6.23, *p* = .006) on gastrocnemius isoleucine concentration. Statistically-significant differences between groups were only detected for MS-*Slc7a5*-KO animals on different protein diets (D**, p<0.01) as indicated. There were significant effects of genotype (*F* (1, 30) = 5.62, *p* = .024) on gastrocnemius glutamine concentration with statistically-significant differences between groups (**, p<0.01) as indicated. (B) There were significant effects of both genotype (*F* (1, 23) = 7.43, *p* = .012) and dietary protein content (*F* (2, 23) = 9.82, *p*<0.001) on plasma isoleucine concentration. Statistically-significant differences between genotype (G*, p<0.05) and dietary protein (D**, p<0.01; D***, p<0.001; wild-type only) groups are indicated. No significant effects on plasma glutamine concentration were detected by 2-way ANOVA.
(TIF)

Figure S5 **Effect of altered dietary protein intake on mTORC1 pathway signalling in MS-** ***Slc7a5*** **-KO mice.** (A) Quantitation of S6K phosphorylation in gastrocnemius muscle normalised to effect of insulin injection for mice of both genders. 2-way ANOVA shows significant effects of both genotype (*F* (1, 54) = 4.76, *p* = .033) and dietary protein content (*F* (2, 54) = 4.50, *p* = .016) on S6K phosphorylation. Statistically-significant differences between genotype (G**, p<0.01) and protein diet (D*, p<0.05; D**, p<0.01; wild-type only) groups are indicated. (B) Quantitation of S6K phosphorylation in soleus muscle normalised to effect of insulin injection for male mice (mean ± SEM for n = 6–7 (WT) and 4–5 (MS-*Slc7a5*-KO) mice. 2-way ANOVA shows significant effects of both genotype (*F* (1, 27) = 16.7, *p* = <.0001) and dietary protein content (*F* (2, 27) = 8.02, *p* = .002) on S6K phosphorylation. Statistically-significant differences between genotype (G*, p<0.05, G**, p<0.01) are indicated.
(TIF)

Figure S6 **Plasma insulin concentration in MS-** ***Slc7a5*** **-KO mice.** (A) Plasma insulin concentrations in mice on 10, 20 and 30% protein diets at time of tissue sampling (fed state). (B) HOMAR-IR values for wild-type (n = 33) and MS-*Slc7a5*-KO (n = 12) mice after 8 h fast. Mean value ± SEM for n = 6–7 (WT) and 4–5 (MS-*Slc7a5*-KO) male mice. No significant effects were detected by 2-way ANOVA. (C) Quantitation of Akt phosphorylation in soleus muscle for male mice on 10, 20 and 30% protein diets.
(TIF)

Figure S7 **Effect of altered dietary protein intake on ATF4 mRNA expression in gastrocnemius muscle of MS-** ***Slc7a5*** **-KO mice.** Mean ± SEM for n = 6–11 (WT) and 4–5 (MS-*Slc7a5*-KO) male mice. 2-way ANOVA detected no significant effects of either genotype or dietary status on ATF4 mRNA levels.
(TIF)

Table S1 **Amino acid concentrations in tissues from heterozygous Bal1-Cre SLC7A5 knockout mice.** No significant differences were detected by unpaired t-test.
(DOCX)
