# Supplementary figures and images for: The Catalytic Subunit of the System L1 Amino Acid Transporter (Slc7a5) Facilitates Nutrient Signalling in Mouse Skeletal Muscle
Source: PLoS One. 2014 Feb 26;9(2):e89547. doi: 10.1371/journal.pone.0089547 (PMC3935884; doi:10.1371/journal.pone.0089547)

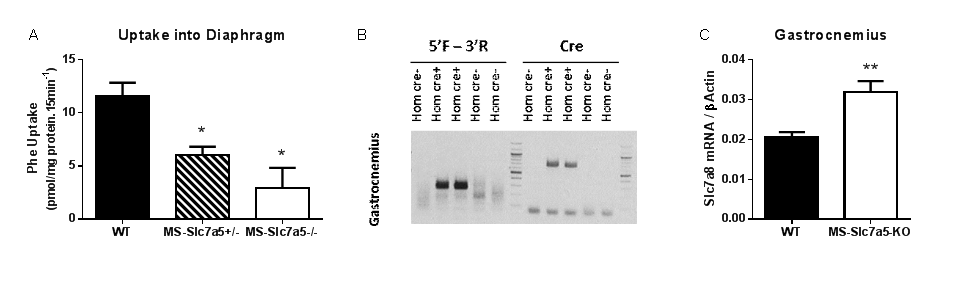

Supplement: Figure S1 — (A) Reduced phenylalanine transport function (measured as Phe uptake) in MCK-Cre-Slc7a5+/− and -Slc7a5−/− mouse diaphragm muscle. Unpaired t-test shows *p<0.05 compared with wild-type. (B) Confirmation of Cre-mediated Slc7a5 gene excision in gastrocnemius muscle of MS-Slc7a5-KO mice. Representative PCR analysis performed on gastrocnemius muscle, using the 9–13 primer pair (5′F-3′R) generates a product of 253 bp only with the recombined Slc7a5 gene lacking the 1855 bp floxed region including exon1. PCR analysis using the 18–19 primer pair (Cre) identifies presence of the Cre recombinase gene. (C) Increased expression of Slc7a8 (LAT2) mRNA in MS-Slc7a5-KO mouse gastrocnemius muscle (n = 17) compared to wild-type (n = 22). **indicates p<0.01 by unpaired t-test. (TIF) [file pone.0089547.s001.tif]

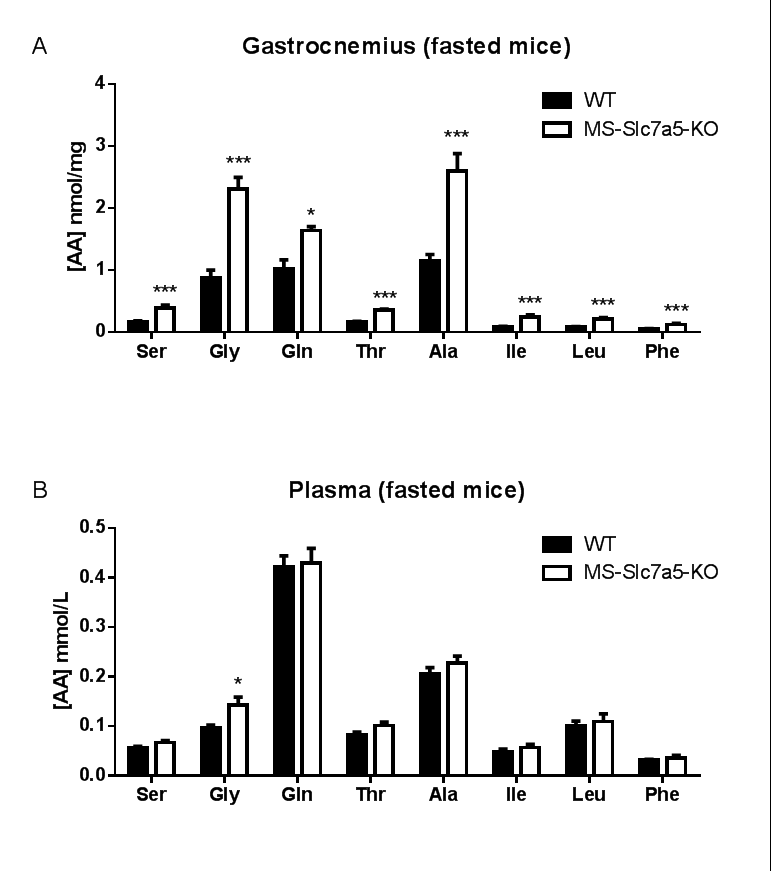

Supplement: Figure S2 — Effect of 8 h overnight fast on muscle and plasma amino acid concentrations in MS- Slc7a5 -KO mice. (A) Intramuscular concentrations of several neutral AA are significantly lower after 8 h fast in WT animals (n = 8) compared to MS-Slc7a5-KO animals (n = 5) (*and ***indicate p<0.05 and p<0.001 respectively by unpaired t-test) (B) Plasma AA concentrations after 8 h fast are broadly similar for wild-type and MS-Slc7a5-KO mice (*indicates p<0.05 for glycine only). (TIF) [file pone.0089547.s002.tif]

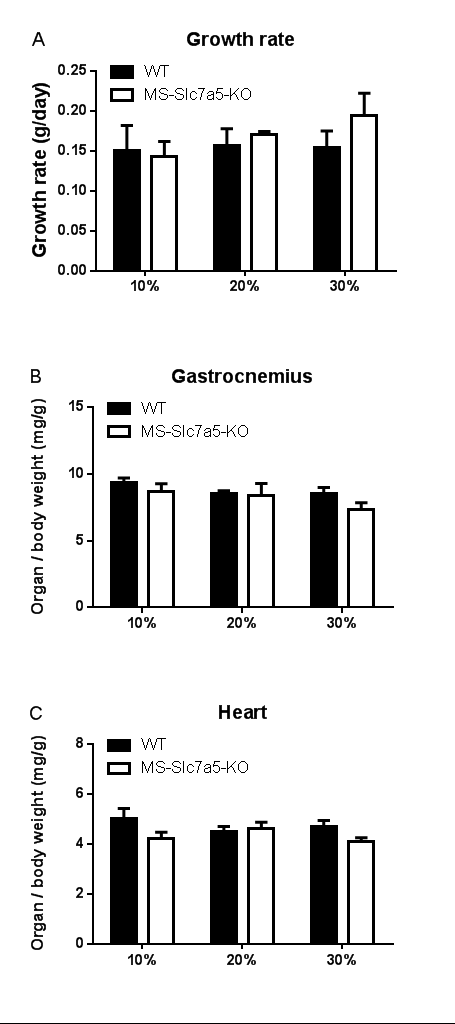

Supplement: Figure S3 — Effect of altered dietary protein intake on growth rate and muscle mass in MS- Slc7a5 -KO mice. Mean value ± SEM for n = 6–7 (WT) and 4–5 (MS-Slc7a5-KO) male mice. (A) Shows the growth rate calculated between days 60 and 80 of age. (b,c) Show the ratio between organ (gastrocnemius (B) and heart (C)) and body weight. No significant effects were detected by 2-way ANOVA. (TIF) [file pone.0089547.s003.tif]

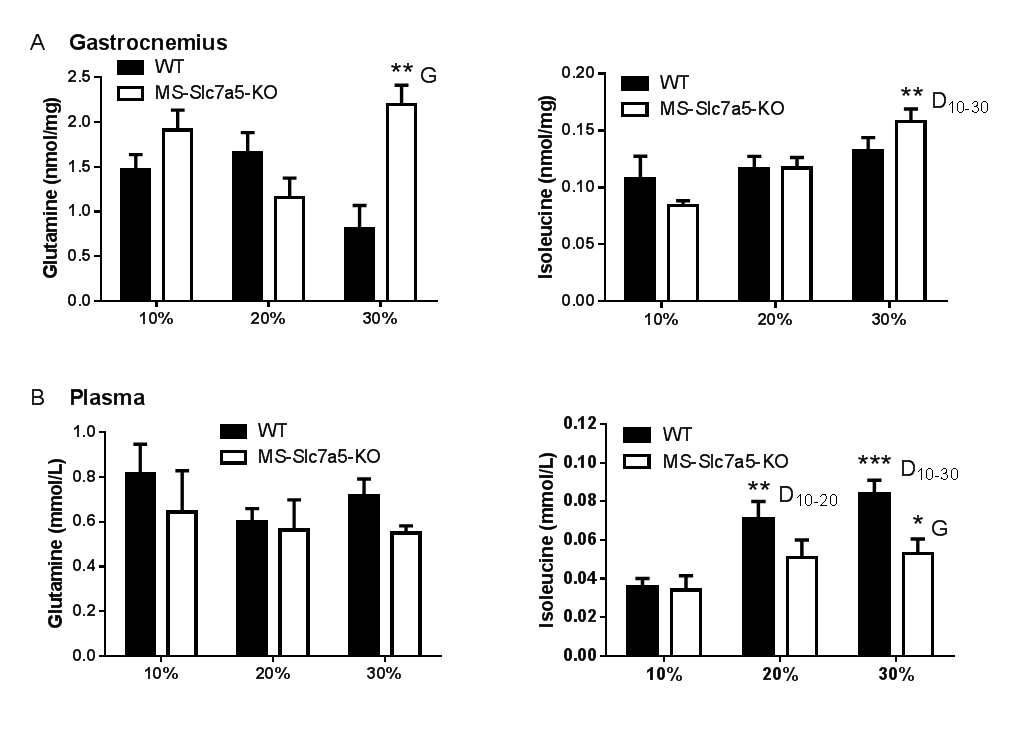

Supplement: Figure S4 — Effect of altered dietary protein intake on muscle and plasma concentrations of isoleucine and glutamine in MS- Slc7a5 -KO mice. Mean ± SEM for n = 6–10 (WT) and 3–5 (MS-Slc7a5-KO) male mice. (A) There were significant effects of dietary protein content (F (2, 27) = 6.23, p = .006) on gastrocnemius isoleucine concentration. Statistically-significant differences between groups were only detected for MS-Slc7a5-KO animals on different protein diets (D**, p<0.01) as indicated. There were significant effects of genotype (F (1, 30) = 5.62, p = .024) on gastrocnemius glutamine concentration with statistically-significant differences between groups (**, p<0.01) as indicated. (B) There were significant effects of both genotype (F (1, 23) = 7.43, p = .012) and dietary protein content (F (2, 23) = 9.82, p<0.001) on plasma isoleucine concentration. Statistically-significant differences between genotype (G*, p<0.05) and dietary protein (D**, p<0.01; D***, p<0.001; wild-type only) groups are indicated. No significant effects on plasma glutamine concentration were detected by 2-way ANOVA. (TIF) [file pone.0089547.s004.tif]

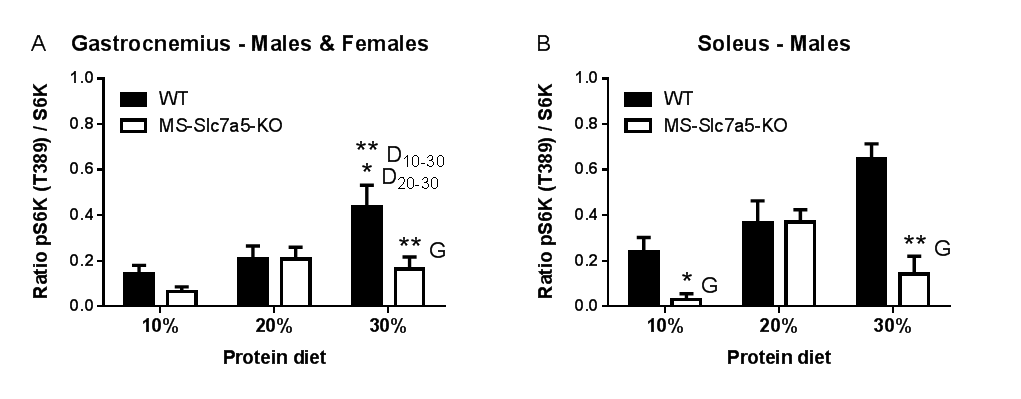

Supplement: Figure S5 — Effect of altered dietary protein intake on mTORC1 pathway signalling in MS- Slc7a5 -KO mice. (A) Quantitation of S6K phosphorylation in gastrocnemius muscle normalised to effect of insulin injection for mice of both genders. 2-way ANOVA shows significant effects of both genotype (F (1, 54) = 4.76, p = .033) and dietary protein content (F (2, 54) = 4.50, p = .016) on S6K phosphorylation. Statistically-significant differences between genotype (G**, p<0.01) and protein diet (D*, p<0.05; D**, p<0.01; wild-type only) groups are indicated. (B) Quantitation of S6K phosphorylation in soleus muscle normalised to effect of insulin injection for male mice (mean ± SEM for n = 6–7 (WT) and 4–5 (MS-Slc7a5-KO) mice. 2-way ANOVA shows significant effects of both genotype (F (1, 27) = 16.7, p = <.0001) and dietary protein content (F (2, 27) = 8.02, p = .002) on S6K phosphorylation. Statistically-significant differences between genotype (G*, p<0.05, G**, p<0.01) are indicated. (TIF) [file pone.0089547.s005.tif]

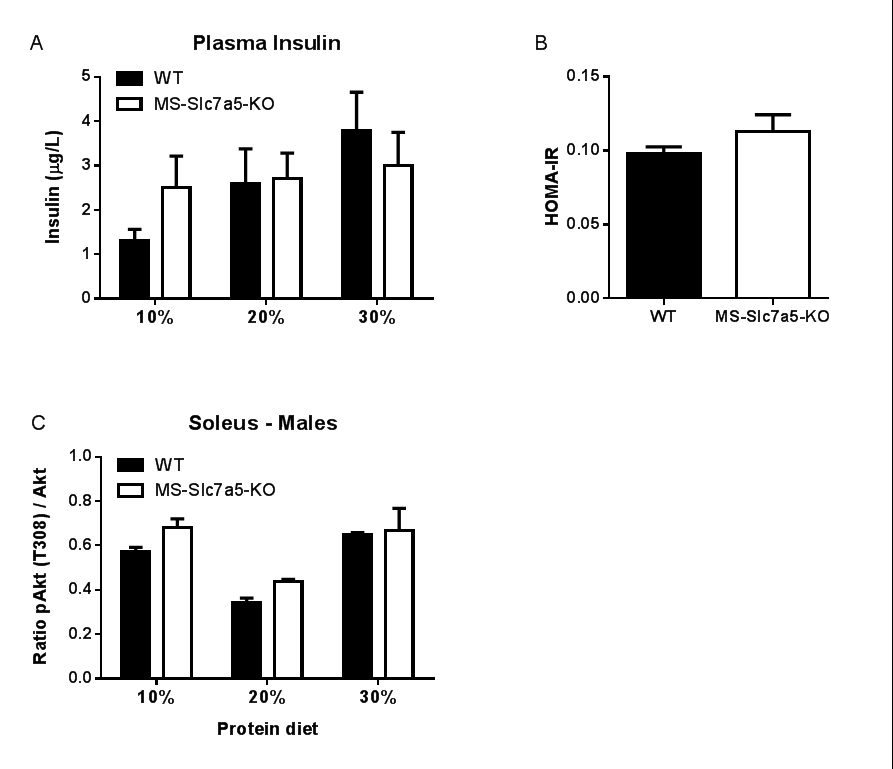

Supplement: Figure S6 — Plasma insulin concentration in MS- Slc7a5 -KO mice. (A) Plasma insulin concentrations in mice on 10, 20 and 30% protein diets at time of tissue sampling (fed state). (B) HOMAR-IR values for wild-type (n = 33) and MS-Slc7a5-KO (n = 12) mice after 8 h fast. Mean value ± SEM for n = 6–7 (WT) and 4–5 (MS-Slc7a5-KO) male mice. No significant effects were detected by 2-way ANOVA. (C) Quantitation of Akt phosphorylation in soleus muscle for male mice on 10, 20 and 30% protein diets. (TIF) [file pone.0089547.s006.tif]

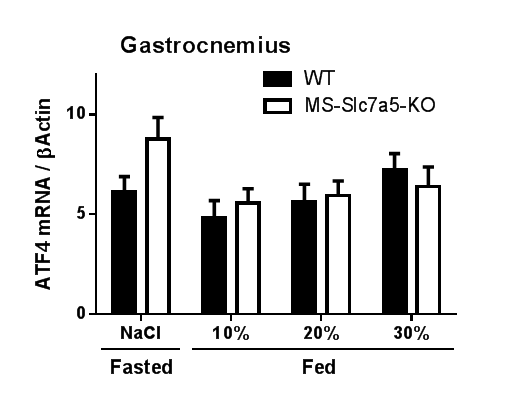

Supplement: Figure S7 — Effect of altered dietary protein intake on ATF4 mRNA expression in gastrocnemius muscle of MS- Slc7a5 -KO mice. Mean ± SEM for n = 6–11 (WT) and 4–5 (MS-Slc7a5-KO) male mice. 2-way ANOVA detected no significant effects of either genotype or dietary status on ATF4 mRNA levels. (TIF) [file pone.0089547.s007.tif]
