# Supplementary material for: The Catalytic Subunit of the System L1 Amino Acid Transporter (Slc7a5) Facilitates Nutrient Signalling in Mouse Skeletal Muscle
Source: PLoS One. 2014 Feb 26;9(2):e89547. doi: 10.1371/journal.pone.0089547 (PMC3935884; doi:10.1371/journal.pone.0089547)
Supplement: Table S1 — Amino acid concentrations in tissues from heterozygous Bal1-Cre SLC7A5 knockout mice. No significant differences were detected by unpaired t-test. (DOCX) [file pone.0089547.s008.docx]

|  | Liver (μmol/g ww) | | Gastrocnemius muscle (μmol/g ww) | | Plasma (mmol/l) | |
| --- | --- | --- | --- | --- | --- | --- |
|  | Slc7a5+/+ | Slc7a5+/- | Slc7a5+/+ | Slc7a5+/- | Slc7a5+/+ | Slc7a5+/- |
|  | n=9 | n=6 | n=8 | n=5 | n=9 | n=6 |
| Glutamic Acid | 1.13 ± 0.21 | 0.817 ± 0.219 | 0.464 ± 0.084 | 0.440 ± 0.096 | 0.059 ± 0.010 | 0.055 ± 0.018 |
| Glutamine | 2.96 ± 0.39 | 2.06 ± 0.33 | 1.97 ± 0.21 | 1.55 ± 0.46 | 0.344 ± 0.041 | 0.400 ± 0.041 |
| Leucine | 0.827 ± 0.098 | 0.651 ± 0.094 | 0.387 ± 0.060 | 0.368 ± 0.110 | 0.145 ± 0.021 | 0.151 ± 0.013 |
| Alanine | 0.535 ± 0.063 | 0.425 ± 0.097 | 2.15 ± 0.29 | 1.91 ± 0.54 | 0.178 ± 0.022 | 0.202 ± 0.021 |
| Serine | 1.77 ± 0.25 | 1.23 ± 0.26 | 0.952 ± 0.123 | 0.612 ± 0.132 | 0.088 ± 0.012 | 0.095 ± 0.006 |

**Table S1**: Amino acid concentrations in tissues from heterozygous Bal1-Cre SLC7A5 knockout mice. No significant differences were detected by unpaired t-test.
